# Review on Load Transfer Mechanisms of Asphalt Mixture Meso-Structure

**DOI:** 10.3390/ma16031280

**Published:** 2023-02-02

**Authors:** Sudi Wang, Weixiao Yu, Yinghao Miao, Linbing Wang

**Affiliations:** 1 National Center for Materials Service Safety, University of Science and Technology Beijing, Beijing 100083, China; 2 School of Environmental, Civil, Agricultural and Mechanical Engineering, University of Georgia, Athens, GA 30602, USA

**Keywords:** asphalt mixture, load transfer mechanism, testing method, contact force, force chain

## Abstract

Asphalt mixture is a skeleton filling system consisting of aggregate and asphalt binder. Its performance is directly affected by the internal load transfer mechanism of the skeleton filling system. It is significant to understand the load transfer mechanisms for asphalt mixture design and performance evaluation. The objective of this paper is to review the research progress of the asphalt mixture load transfer mechanism. Firstly, this paper summarizes the test methods used to investigate the load transfer mechanism of asphalt mixtures. Then, an overview of the characterization of load transfer mechanism from three aspects was provided. Next, the indicators capturing contact characteristics, contact force characteristics, and force chain characteristics were compared. Finally, the load transfer mechanism of asphalt mixtures under different loading conditions was discussed. Some recommendations and conclusions in terms of load transfer mechanism characterization and evaluation were given. The related work can provide valuable references for the study of the load transfer mechanism of asphalt mixtures.

## 1. Introduction

Asphalt mixture is a typical composite material comprising aggregates, asphalt binders, fillers, and voids. The extrinsic uncertainty, irregularity, vagueness, and nonlinearity of asphalt mixtures’ properties (such as mechanical properties) are the reflection of its microstructural complexity. The skeleton structure is regarded as the main body to transfer external loads in asphalt mixture and the aggregate skeleton contributes the most to the high temperature stability of asphalt mixture. Therefore, the performance of asphalt mixtures can be evaluated from the load transfer mechanism of the materials. The study of the load transfer mechanism of the meso-structure of asphalt mixture provides a new perspective for the study of classical problems, and can also induce new understanding. In the past decades, a variety of methods have been introduced to study the load transfer mechanisms, such as the photoelastic experiment method, digital speckle method, digital image processing (DIP) technology, discrete element method (DEM) simulation, finite element method (FEM) simulation, and the combination of multiple methods [1,2,3,4,5,6,7,8,9,10,11,12].

The load transfer mechanism mainly refers to the characteristics of load transfer in an asphalt mixture under external load [5,13]. Aggregates in contact constitute the skeleton of asphalt mixtures and affect load transmission in the mixture, which determines its deformation resistance [14,15]. Meanwhile, the load transfer characteristics can be used to predict the load-bearing capacity of the aggregates and asphalt mixtures. Recent studies of the load transfer mechanism in asphalt mixture focus on three aspects: contact mechanical behavior, contact force characteristics, and force chain characteristics. The research on contact mechanical behavior of asphalt mixture is mainly focused on analyzing the aggregate contact, such as aggregate contact zone [16], aggregate contact chain structure [2,17,18,19], and aggregate contact number characteristics [20,21]. For contact force characteristics, the most common studies are statistical analysis of contact force, including contact force distribution characteristics [9,22,23] and contact force evolution characteristics in the compaction process [5,24]. Studies of force chain fall into two categories, force chains identification criteria, including force chains length [25,26] and force chains number [27,28], and force chains structural characteristics [6,29]. From the existing studies, it is found that the contact skeleton structure of asphalt mixture is the load transfer foundation, and the contact force and force chain characteristics can reflect load transfer mechanism of skeleton structure of asphalt mixtures. Meanwhile, massive research attention has been given to develop the evaluation parameters of load transfer mechanisms according to load transfer characteristics, such as contact number, contact chain structure, load-bearing contributions (based on average contact force), and force chains morphologies (force chains length, force chains angle, and force chains number) [5,16,21,28].

There are different load transfer mechanisms of asphalt mixtures under different test states, from compaction to service to destruction. The common compaction methods are the Marshall impact compaction (MIC) method [30], rotary compaction experimental method [31], and field compaction [32,33]. Exploring the load transfer mechanisms in different compaction processes provides references for evaluating the stability of the load-bearing structure of asphalt mixtures after compaction. Meanwhile, the load transfer mechanism of asphalt mixtures under different external load conditions, such as tension–compression conditions [34], shear conditions [35], and bending conditions [36], is the mechanical response of asphalt mixture to different field service conditions, which can provide the mechanical explanation of the macro-scale damage of asphalt mixture from the meso-scale mechanical mechanism.

**Figure 1:**
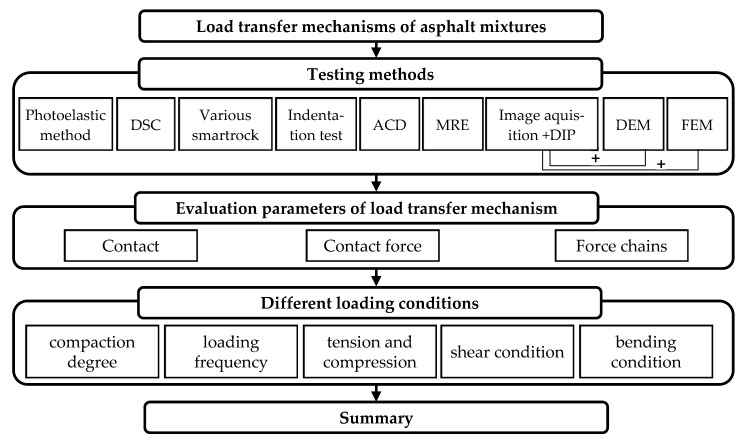
Flowchart of this paper. Note: DEM—Discrete element method; FEM—Finite element method; DIP—Digital image processing; DSC—Digital speckle correlation; ACD—Aggregate contact device; MRE—Magnetic Resonance Elastography; Various smartrock—includes Intelligent Aggregate, Intelligent Attitude Aggregate, and SmartRock.

The objectives of this review are the following, and the flowchart of this paper is shown in Figure 1.

(1) Present the comprehensive review of the test methods and techniques for the load transfer mechanism of asphalt mixtures.
(2) Collect and discuss the characteristic parameters of the load transfer mechanism.
(3) Compare the load transfer mechanism of asphalt mixtures under different loading conditions including compaction degree, different loading frequency, tension–compression, shear condition, and bending condition.
(4) Provide recommendations to select and improve the load transfer characteristics and evaluation parameters of the load transfer mechanism in asphalt mixtures.

This paper mainly reviews and summarizes the meso-scale load transfer (mainly referring to contact structure, contact force, and force chain) and related evaluation indicators to provide a theoretical reference for the investigation of the macro-scale properties of asphalt mixture at the level of meso-scale mechanical mechanisms.

For this review, the search papers across the three databases, Web of Science (WoS), Scopus, and China national knowledge infrastructure (CNKI), were used. The keywords searched mainly include asphalt mixture, meso-scale structure, load transfer, contact, contact force, force chain, pavement, aggregate blend, DEM, and FEM, etc. Most of the selected articles in this paper are published as journal articles.

## 2. Definitions and Test Methods for Load Transfer Mechanism

### 2.1. Definition

**Figure 2:**
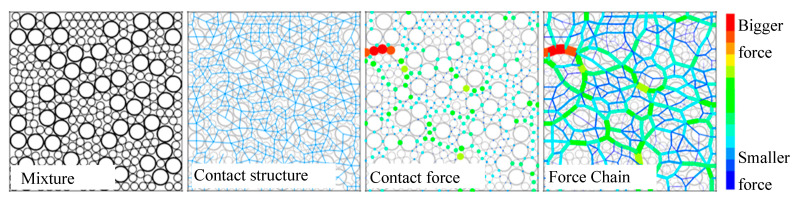
Load transfer characterizations of asphalt mixture: contact (blue line), contact force (colorful sphere), force chain (colorful line).

For the load transfer mechanism, the most common studies fall into three categories: contact [3], contact force [5], and force chain [13]. A schematic diagram is shown in Figure 2. The corresponding concepts are defined as follows.

(1) Contact: This reflects the contact information between particles in asphalt mixtures. The contact skeleton of load transfer is formed between particles. Typically, the total contact number in asphalt mixtures can be defined by Equation (1) [5].

(1) $$\left[\begin{array}{cccc} C_{m\sim m} & C_{m\sim \left(m+\Delta s_{1}\right)} & \cdots & C_{m\sim M}\\ & C_{\left(m+\Delta s_{1}\right)\sim \left(m+\Delta s_{1}\right)} & \cdots & C_{\left(m+\Delta s_{1}\right)\sim M}\\ & & \ddots & \vdots \\ & & & C_{M\sim M} \end{array}\right]=C$$

where *C_i~j_* represents the contact number between particles of *A_i_* and *A_j_*, *m* is the smallest particle size in the aggregate blend, *M* is the largest particle size in the aggregate blend, and $\Delta S_{i}$ is the gap between adjacent sieve sizes; *i* represents the particle size, *i* = 1, 2, …, *M*.

(2) Contact force: This represents the force transferred between particles through each contact point.

(3) Force chain: This presents the path of transferring external loading in asphalt mixtures and consists of the contact between particles and contact force.

### 2.2. Test Methods and Comparative Evaluation

**Figure 3:**
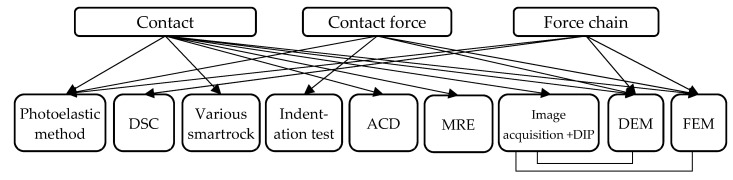
The relationship between test methods and load transfer mechanism characteristics: Various smartrock includes Intelligent Aggregate, Intelligent Attitude Aggregate, and SmartRock.

There is no standardized test method to assess the load transfer mechanism of asphalt mixtures. According to the previous study, the test methods of load transfer mechanism characterizations can be divided into simulation methods and laboratory test methods. The basic information regarding the corresponding methods to investigate the load transfer mechanism is presented in Table 1. The relationship between the various test methods and the types of load transfer mechanisms is shown in Figure 3.

For the laboratory test methods, the use of image acquisition and DIP to analyze the contact characteristics of asphalt mixtures is more accurate [8,17,37], but the test workload and expenses are large. Furthermore, the load transfer mechanism cannot be well characterized. The photoelastic test can be used to analyze both contact characteristics and contact forces as well as the force chain [38,39,40,41]. However, there are high material property requirements and cost limitations for the photoelastic particles, and the photoelastic test limits the study to 2D. The digital speckle correlation test is applicable to the analysis of strain variation of the specimen cross-section [42,43]. Various smartrocks, including Intelligent Aggregate [44], Intelligent Attitude Aggregate [46], and SmartRock [33,45], are only applicable to a point in the asphalt mixture, and the test results have limitations due to the relatively few collected data. The indentation test can be used to characterize the magnitude and distribution of contact forces on a single contact surface, but it cannot characterize the load transfer in the whole blend structure. The Aggregate contact device (ACD) equipment can be used to interpret the relationship between contact structure changes and energy dissipation during compaction, while it cannot be used to characterize load transfer [48]. For the Magnetic Resonance Elastography (MRE) method, the contact structure of the mixture under external loading can be imaged using the MRE pulse sequence [49]. The application of the MRE method requires high technical requirements, and the contact structure obtained has certain errors because the data processing accuracy cannot be reached. For the numerical simulation test methods, such as the finite element method [2,3,50,51] and discrete element method [5,7,28], it is more convenient to analyze the influence of a single factor and to exclude the cross influence of many factors on the load transfer mechanisms. It can also reduce time consumption and cost, using the numerical simulation test method. The model parameters or material characteristics must be determined, but this can be challenging. Generally, the model parameters or material properties used in the aforementioned studies were obtained through reverse modeling rather than directly from experimental data. From the above analysis, there are some limitations when laboratory test methods are used to characterize certain load transfer mechanisms. Numerical simulation tests are more suitable for characterizing the load transfer mechanism of asphalt mixtures. Furthermore, the combination of DIP and a numerical simulation method, which is the recommended method to be used in current and future research, can provide a better way to investigate the load transfer mechanism of an asphalt mixture [52,53]. Based on the meso-scale structure of asphalt mixture obtained by the DIP method, the asphalt mixture load transfer characteristics can be studied by a numerical simulation. It should be noted that for the study of load transfer characteristics, there is no feasible method to obtain the load transfer (contact structure, contact force, or force chain) in real three-dimensional mixtures.

## 3. Characterization of Load Transfer Mechanism

This section provides a generalized overview of the characterization of contact, contact force, and force chain. The typical evaluation indicators are also illustrated and compared as follows.

### 3.1. Contact Characteristics

Aggregate contact characteristics, including the contact number, contact distance distribution, contact length distribution, and contact orientation, etc., are descriptions of the adjacent aggregate particle interaction, which play an important role in the load transmission in asphalt mixtures. The studies of contact in asphalt mixtures fall into two categories: (1) parameterization of aggregate contact [20,30,54,55,56,57] and (2) contact chain characterization [58,59,60,61]. The parameters such as aggregate contact point, average contact length, total contact length, and contact orientation can be obtained from a section image of the asphalt mixture. The contact chain network in an asphalt mixture is a complex topological structure, which changes with the change of external load action [30,62,63,64,65].

Initially, contact points are usually used to describe contact characteristics and evaluate the meso-structure performance of asphalt mixtures [56]. However, several researchers found that the contact points cannot fully judge the quality of the mixture structure [20,66]. With the development of computer technology, some other contact indicators are developed to distinguish the contact structure of different mixtures using an image processing method. Jiang et al. [21] established a series of contact structure indicators in respect of contact distance distribution, contact length distribution, and contact orientation by using a two-dimensional image acquisition and processing program. They found that the optimal contact distance threshold for contact line analysis is 0.5 mm. The contact length distributions are varying in different mixtures. Kutay et al. [61] proposed a calculation method for the aggregate contact point and direction in hot mixture asphalt (HMA) using image processing technology based on CT images, and analyzed the change of contact characteristics with increasing compaction degree. The results show that the number of contact points increases significantly with the increase of compaction degree. Cai et al. [67,68] characterized the contact characteristics of an asphalt mixture under different compaction cycles by introducing indicators such as coarse aggregate contact point and aggregate inclination based on digital image processing technology. The results show that when the compaction force exceeds the limit of the skeleton bearing capacity, the contact point decreases and the inclination of the aggregate fluctuates. With the increase of compaction repetitions, the average contact number increases first and then decreases. Xing et al. [69] proposed a calculation method for aggregate classification and contact performance, discussed the impact of failure on meso-structure and aggregate contact, and analyzed the relationship between disruption factor and contact characteristics based on X-CT and digital image processing technology. It was found that a higher disruption factor could reduce the number of aggregate contacts in the main skeleton and increase the number of contacts of broken aggregates. Shi et al. [17,19,70,71] characterized the contact chains in asphalt mixture using DIP. The parameter of modularity according to the spectral clustering method was used to evaluate the contact chains [72]. The skeleton mechanical behavior can be improved by obtaining a maximum amount of coarse aggregate in the contact chains. In conclusion, the indexes such as contact length and aggregate inclination obtained by digital image technology are able to well describe the contact characteristics. However, image processing technology can only analyze the acquired images and it is time-consuming and laborious.

DEM is a useful method for examining the meso-mechanical characteristics of granular materials and is crucial for understanding the meso-structures of asphalt mixtures. The contact properties of asphalt mixtures based on DEM have been the subject of numerous studies, including those on the impact of coarse aggregate morphology on the mechanical properties of the skeleton [34,73,74,75], contact meso-structure evaluation indices [76], and the impact of the contact skeleton on impairing the movement of coarse aggregates [77,78]. The volume indices, rutting resistance, durability, and road performance of asphalt mixtures are all positively correlated with the meso-scale properties of aggregate contact [79]. The contacted coarse aggregate is what makes up the contact chains in asphalt mixture, which together form a complex network that affects the macro-mechanical characteristics of the asphalt mixture. These contact chains operate as a bridge between the micro- and macro-scale properties of materials. Qian et al. [30] analyzed the influence of different compaction methods (Marshall impact compaction and static compaction) on the distribution characteristics of contact number with depth of specimen using DEM simulation test method. Tan et al. [16] established 3D FEM models based on CT scanning images by incorporating AC cores into the numerical model and quantified the impact of aggregate contact zone ratio on the visual properties of matrix phase. It was discovered that although the contact zone only makes up a minuscule volume proportion of AC, due to its substantially greater modulus than asphalt matrix, it can significantly raise the modulus of AC within the low-frequency region. After conducting a number of studies, Jin et al. [2,3,4,18,80] proposed a novel method based on graph theory for the prediction and evaluation of mixture stability. This method characterizes and assesses the initial and evolutionary morphologies of 3D aggregate contact chains during simulations and offers a significant new direction for the study of asphalt mixture contact chains.

To sum up, the contact skeleton structure formed by the particles in contact with each other is the load transfer path. The internal force transfer is the key to reveal the effect of the contact structure on mechanical properties. Then, it is essential to obtain the internal contact force response of asphalt mixtures to investigate the load transfer mechanism.

### 3.2. Contact Force Characteristics

In many studies, the contact force between particles refers to the normal component [5,7,9,22]. Generally, contact forces are classified as strong and weak, and the strong constitutes the main load-bearing system in asphalt mixtures. Initially, it is considered that the contact forces between coarse particles form the strong and the contact forces between fine particles constitute the weak contact forces [81]. With the development of numerical simulation techniques, the values of contact forces can be extracted, which promotes the quantitative studies. It is common to define contact forces greater than the average as strong and those less than the average as weak [6,22].

Due to the anisotropy of the contact force distribution, there is a certain deficiency to characterize the contact force distribution by the strong or weak alone. Therefore, a series of studies are carried out to characterize the contact force distribution, mainly including two categories: contact force probability distribution [82,83] and contact force statistical characteristics [23,28,84,85]. Shashidhar and Shenoy [41] studied the contact force distribution in asphalt mixtures by means of a photoelastic experiment. The results show that different gradations exhibit different contact force distribution characteristics. Jiang et al. [83,86] explored the contact force distribution in the tight arrangement of single size particles by indentation experiments. The contact force of each layer particles is detected by the indentation of the impress board. It was found that the contact force probability distribution is approximately parabolic. Chang et al. [9] studied the contact force probability distribution characteristics for different grain size compositions using the indentation test. It was found that the probability of contact force distribution decayed exponentially for all different two-size compositions. To further explore the contact force distribution within different asphalt mixtures, Chang et al. [22,23,87] compared the contact force probability distribution of three type asphalt mixtures using DEM, Stone Matrix Asphalt (SMA) gradation, dense asphalt concrete (AC) gradation, and open-graded asphalt friction course (OGFC) gradation with a nominal maximum aggregate size (NMAS) of 13.2 mm. It was found that for the three type asphalt mixtures, the probability distributions of the normal contact forces show no significant difference. The probability of contact forces (*P(f)*) decreases with the increase of *f_n_* (the normal contact force to the mean normal contact force) when *f_n_* ≤ 0.75. When 0.75 < *f_n_* ≤ 1.65, *P(f)* is directly proportional to *f_n_*, and when *f_n_* > 1.65, *P(f)* is inversely proportional to *f_n_*; *P(f)* remains essentially unchanged at *f_n_* ≥ 4.

Some researchers also used various statistical parameters to characterize the features of contact forces. Zhu et al. [62] defined the vertical contact unbalanced force, which is calculated as the sum of the contact force vectors and the gravity of aggregate. It was found that the larger the particle size, the more the contact number, the greater the contact unbalance force in the Marshall impaction process. Generally, certain size particles have different load transfer characteristics in different grain size compositions. Zhang et al. [79] studied the contribution of each size aggregate to forming a skeleton structure by contact force analysis. The force occupation contributing to the formation of the aggregate skeleton is defined as the ratio of the contact force bigger than the total average force in one sieve size to the contact force larger than the total average force in all sieve sizes. The force extraction analysis demonstrated that, regardless of the total number of particles in the various sieve sizes, the bigger size included more contact force in each particle. It was found that 2.36 mm and 4.75 mm, which together contribute more than 50% of the main load carrying capacity, are the key sieve sizes in the primary structure. While 0.3 mm to 1.18 mm, which also contributes more than 50%, is a crucial sieve size range for stabilizing the basic structure. A series of DEM tests were conducted by Miao et al. [7] to examine the contact force characteristics of various sized particles in aggregate blends. To characterize the load-bearing contribution of each size particle in aggregate blends, an indicator was suggested. Wang et al. [5] also used DEM to examine the load-bearing contributions of various aggregate blends while taking into account the morphology of the aggregates. The critical load-bearing contribution particle for the SMA16 gradation was discovered to be 2.36 mm, while for the AC25 gradation, it was found to be 4.75 mm.

From the above studies, a lot of research has been carried out on the characterization of contact force distribution. The contact force distribution can only explain the overall force state of the asphalt mixture, but it cannot fully reveal how the load is transferred in the asphalt mixture and whether the load is transferred uniformly. The composition of asphalt mixtures has an influence on the contact force distribution, and the load-bearing capacity of each size aggregates also has effects on the load transfer mechanism. Based on current studies, the contact force characteristics of asphalt mixture need to be further analyzed.

### 3.3. Force Chain Characteristics

Numerous studies have found that the discontinuous and non-uniform arrangement of particles forms complex contact networks, which is the load transfer path [64,88]. However, the force chain is conceptually different from that of the contact network. The force chain is a selective force transfer path along the contact network, while the contact network is a geometric structure with granular particles in arrangement. The force chain is extremely sensitive to the loading method and the geometric characteristics of the system. Even in the same contact network, a slight change in the external loading can make the force chain very different. Dantu [89] specified the non-uniformity of force distribution inside the particles in the photoelastic experiments. It was found that the force chain has a tree-like structure. Edwards and Oakeshott [90] found the force chains arching in granular blends in 1989. Then, Bouchaud and Cates [91] further studied the force chain and explicitly introduced the concept of force chains.

Initially, laboratory experimental methods were used to study force chain characteristics in granular materials. He et al. [92] utilized the photoelastic method to study the load transfer in the asphalt mixture. Wang et al. [93] obtained the evidence of structural transformation of force chains under shear vibrations using mechanical spectroscopy. Sanfratello et al. [49] used magnetic resonance elastography (MRE) to observe and describe the three-dimensional force chain in granular materials. Generally, the main drawback of the experimental method is the inability to detect weak contact forces and the inability to detect the contact forces inside the blend without interference. Using the DEM, the force chain can be elaborately characterized. Sun et al. [64] studied the load transfer characteristics of granular blends under uniaxial compression by means of 2D DEM, proposed the angular criterion of the force chain, and found that the length of the force chain is distributed by the power rate. Zhang et al. [94,95] quantified the force chain characteristics during the high-speed compression of granular blends. It was found that the higher the initial impact velocity, the more the number of force chains, and the shorter their length. Additionally, the force chain direction showed anisotropy and formed an irregular distribution.

The mechanical characteristics of granular materials are influenced by force chains, which are a key component of the granular material mechanics theory [96]. Several studies furthering skeletal contact force statistical analysis try to assess the force chains in asphalt mixes. By using DEM, Chen et al. [81] qualitatively investigated the force chains in crumb rubber asphalt mixtures and categorized them only according to contact force magnitude. Based on a CT scanning picture, Wang et al. [97] created a 3D FEM model of an asphalt mixture that showed how internal load transmission develops in asphalt mixes. It was discovered that the aggregates bear the highest stress and that force chains build practically along their skeleton. Shi et al. [98] presented aggregate contact point efficacy parameters and evaluated force chains in SMA13 and AC13 asphalt mixes. These are all qualitative evaluations, which have limitations in terms of disclosing the properties of force chains. Chang et al. [23] developed force chain direction angles to evaluate asphalt mixture force chains morphological characteristics, and discovered asphalt mixture internal loading transfer law in order to quantitatively assess asphalt mixture force chains. In addition, Liu et al. [85] constructed asphalt mixed force chains by concurrently taking into account granular quantity and contact angle. According to the findings, the performance of various aggregate mixes can be reflected in considerable force chain differences. Liu et al. [82] evaluated the force chains number of dense-suspended and dense-skeleton asphalt blends based on the aforementioned force chains identification criteria. Additionally, systematic analysis of the asphalt mixture force chains identification criteria of Liu et al. [84] revealed that the suggested threshold values for contact force and angle are the average contact force and 45°, respectively. Based on known force chains identification criteria, Liu et al. [13] looked into the length distribution of force chains. The findings indicate that raising NMAS can contribute in the formation of force chains in asphalt mixes that are longer in length.

The study of force chains can systematically reflect the load transfer mechanism of the asphalt mixture, which effectively avoids the limitation of evaluating the overall load-bearing capacity due to the unilateral analysis of contact or contact force. However, the evaluation characterization of force chains has not formed a completed system, and the characterization of force chains is only at the stage of basic statistical analysis.

### 3.4. Comparison of Different Indicators

According to previous studies, a series of indicators have been proposed to characterize the load transfer mechanisms of asphalt mixtures. The typical indicators for load transfer quantitative characterization are summarized in Table 2. The indicators of contact characteristics can better characterize the contact skeleton structure and evaluate the goodness of the contact geometry structure. However, it cannot explain the force state in asphalt mixtures. The contact force characteristics can be used to characterize the overall force state of asphalt mixtures, to determine the overall load-bearing structure, and to evaluate the load-bearing capacity of each aggregate size. However, the contact force characteristics are only a statistical analysis of the contact forces at all contact points and do not provide an assessment of the load-bearing capacity of the load transfer structure. For the indicators of force chain characteristics, they can characterize the load transfer paths in asphalt mixtures and the force state of the contact structure.

Based on the review reported above, the existing parameters are insufficient for investigating the characteristics of the load transfer mechanism of each particle size. The contact structure is a complex topological structure where contact forces are transferred at various nodes to form force chains. Through the above research summary, no definite quantitative index is given to clearly define the load transfer characteristics. Meanwhile, it does not reveal the essential issues of grade design, material selection, and service performance quality of asphalt mixtures from the level of load transfer mechanism. Further studies are still needed to be carried out to combine the contact, contact force, and force chains, and thus, to reveal the relevant mechanical mechanisms in depth.

## 4. Load Transfer Mechanism of Asphalt Mixture under Different Loading Conditions

There are different load transfer mechanisms in asphalt mixtures from compaction to service to destruction. Many researchers have conducted studies to analyze the load transfer mechanism of asphalt mixtures under different test states. Firstly, the load transfer mechanisms of asphalt mixture during the compaction process are summarized. Then, the internal load transfer mechanism of asphalt mixture under different test conditions is introduced. A comparative analysis of the load transfer mechanism under different test conditions is also carried out.

### 4.1. Load Transfer Mechanism in the Compaction Process

In the process of asphalt mixture compaction, with the application of external load and different load frequency, the spatial position of aggregate changes [78], then the load transfer characteristics in the contact structure, are changed. Exploring the changes of external load transfer characteristics in different compaction processes provides an important reference basis for evaluating the goodness of the load-bearing structure of an asphalt mixture after compaction.

The load transfer characteristics in compaction are usually investigated by simulating laboratory experiments. The Marshall impact compaction (MIC) method is most commonly used for fabricating asphalt mixture specimens. However, it was found that the load transfer characteristics inside the specimen under double-sided Marshall impact compaction and single-sided Marshall impact compaction are different [30]. The middle part has a large number of contacts, and the two sides have a small number of contacts. The contact number during double-sided compaction is more uniform than it is under single-sided compaction. In order to assess the variance in load transfer during the Marshall impact compaction process of asphalt mixes, Zhu et al. [62] employed the vertical contact unbalanced force. Using DEM modeling, it was possible to measure the vertical contact imbalanced force and the contact number for compaction numbers of 2, 4, 6, 8, 10, 15, 20, 25, 30, 35, 40, 45, 50, 55, 60, 70, 80, 90, and 100 [62]. It was discovered that when the number of strikes increased, the imbalanced force of each particle size progressively reduced. With each increase in strikes, the force of the contact imbalance decreased. The contact imbalance force in the MIC process increases with aggregate particle size, coordinate number, and contact unbalance. As the NMAS of the aggregate is larger, the poorer the Marshall compaction effect. In order to make the compaction process closer to the roller compaction in field, more attention is paid to fabricate the specimen using the rotary compaction method. Gong et al. [74,78] took both gyration angle and rotation action into account and investigated the displacement variation of aggregates in the compaction process. Miao et al. [7] investigated the contact force distribution and transfer characteristics of asphalt mixtures in rotary compaction using DEM. Different gradation asphalt mixtures have different contact force distribution characteristics, under the same external load, different size aggregates have different average contact force. The contact force evolution of different asphalt mixtures during compaction is also different [5]. Liu et al. [31] studied the mechanisms of aggregate movement and contact force changes within asphalt mixtures during a simulated compaction test. The results showed that the contact forces are mainly generated between aggregates.

The load transfer in the compaction process of laboratory specimen cannot completely reflect that of actual pavement in the field. Therefore, it is necessary to establish the relationship between field compaction and laboratory compaction for understanding the load transfer mechanism of asphalt mixtures. Dan et al. [32,33] designed a field test program and used SmartRocks to measure the load transfer response during vibrating compaction. Meanwhile, the load transfer of asphalt mixture during different gyratory compaction degree was also analyzed. It was found that the gyratory compaction degree and the peak acceleration of the vibration drum exhibit a strong linear correlation. By controlling the gyratory compaction degree of asphalt mixtures, the load transfer mechanism in the actual pavement compaction process can be better simulated.

### 4.2. Load Transfer Mechanism under Different Loading Conditions

According to previous studies, there are different test conditions for the study of the load transfer mechanism of asphalt mixtures. Under different external loads and dynamic loading frequencies, the load transfer response in the asphalt mixture is different. Furthermore, the contact forces in the top part of the sample are always higher than those in the lower part [99]. Chang et al. [23] investigated the contact force probability distribution of asphalt mixtures under haversine loading. It was found that the probability distributions of smaller contact forces are greater than that of larger contact forces, and the probability distribution of larger contact forces is the largest when the ratio of contact force to mean contact force is 1.75. Considering the actual vehicle loads, some researchers have studied the load transfer mechanism of asphalt mixtures under uniaxial loading and biaxial loading. Liu et al. [28,82] investigated the load transfer characteristics of AC, SMA, and OGFC under the single-wheel pressure surface load. There are different load transfer characteristics of different gradations of asphalt mixtures. The external loads were mainly transferred along the vertical direction, although a small amount of loads tended to extend horizontally. Meanwhile, the load transfer characteristics between the different structural layers of the pavement have an important influence on the whole structure’s load-bearing capacity. The contact force distribution in high-modulus asphalt concrete (HMAC) pavement structure after double circular static loading was studied [100]. It was found that the application of HMAC decreased the vertical force in all structural layers except the upper surface layer, and the HMAC decreased the horizontal force in the subbase layer.

**Figure 4:**
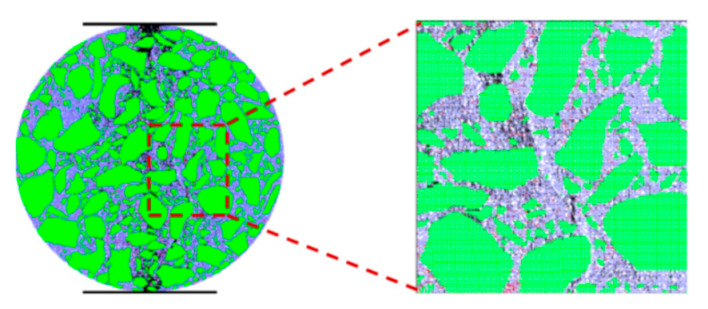
The indirect tensile (IDT) test of asphalt mixtures [52]: the green balls represent aggregates; the blue balls represent mastic; the white balls represent air voids; the black line represents the force chain.

The tension–compression conditions, shear conditions, and bending conditions are the typical loading conditions of asphalt pavement [101]. Ma et al. [34] constructed a virtual tracking test model for asphalt mixtures and analyzed the micro-mechanical response of load transfer in the asphalt mixture under compression conditions. The results showed that contact forces primarily exist underneath the loading pressure area. Under the virtual wheel tracking test, Xue et al. [102] investigated the load transfer characteristics for different gradations of asphalt mixtures, including AC13 and SMA13. The average contact force increases continuously during the loading time from 5 min to 60 min. It was also found that the average contact force between coarse aggregates was the largest, followed by the average contact force between aggregate–mastic and the average contact force between mastics. Peng and Sun [103] used image analysis and DEM to simulate the indirect tensile (IDT) test of asphalt mixtures under tension–compression conditions (shown in Figure 4). The contact force distribution at microcracks was analyzed. Under the vertical loads, the contact forces exhibit compression and tension along the vertical and horizontal directions, respectively.

Chen et al. [35] utilized the DEM numerical simulation penetration test to explore the contact force characteristics of aggregate particles under shear conditions. It was found that under the same penetration depth, the average contact force of the larger size aggregates is greater than that of the smaller size aggregates, and the contact force proportion taken by different aggregates depends on aggregate sizes. The larger the aggregate size, the more proportion of the contact force. Peng and Sun [104] simulated the uniaxial penetration test of asphalt mixtures using DEM. Ding et al. [75] analyzed the load transfer response of AC13 and SMA13 asphalt mixtures in virtual penetration tests. It is known that the contact forces of AC13 and SMA13 were mainly distributed in 0–5 N, accounting for 75–85%. The bigger the contact force, the smaller the corresponding probability distribution proportion.

A random heterogeneous DEM model was employed by Xue et al. [36] to simulate the semi-circular bending (SCB) test of asphalt mixes. It showed that, prior to cracking, tension contact forces were primarily focused in the notch tip and compression contact forces were primarily concentrated in the specimen’s top and bottom. After the specimen cracked, the tension contact force concentration zone traveled from the fracture tip to the top of the specimen over time, but it was always there. It was believed that the primary cause behind crack propagation was the tension force.

From above review, the researchers have studied the load transfer characteristics of asphalt mixtures under different test conditions from various loading aspects. It is indicated that the load transfer mechanism of asphalt mixtures under different test conditions are not the same. Although the analysis of the load transfer mechanism was carried out in the mentioned studies, only a preliminary statistical analysis of contact force distribution characteristics under the corresponding test conditions were carried out. There is no relevant evaluation system of the load transfer mechanism under different loading conditions established. The meso-scale load transfer mechanism under different loading conditions is the mechanical response of the macro-scale properties of the asphalt mixtures. It is necessary to carry out further relevant studies to investigate load transfer mechanisms of asphalt mixtures and establish the corresponding evaluation system of the load transfer mechanism, which will provide a theoretical basis for explaining the macro-mechanical properties from the meso-scale mechanical mechanism.

## 5. Conclusions

Quantitatively capturing the relationship between the load transfer mechanism and the mechanical response of asphalt mixtures can provide a meso-mechanical basis for optimizing asphalt mixture design to improve the performance of asphalt pavement. This paper reviews the research progress of the load transfer mechanism of asphalt mixtures. Some conclusions are drawn as follows.

(1) The study of the load transfer mechanism consists of three main aspects: contact characteristics, contact force characteristics, and force chain characteristics. Various technical methods for studying the load transfer mechanism are summarized and comparatively analyzed. With the comprehensive analysis of different methods used in characterizing the load transfer mechanism, the X-ray CT and DIP and numerical simulation is highly recommended to be used for investigating the load transfer mechanism of asphalt mixtures.

(2) A systematic summary analysis of load transfer mechanism evaluation indexes revealed that the application of several evaluation indicators in combination could be better for characterizing load transfer mechanisms, and the statistical methods can obtain better typical quantitative indicators.

(3) The meso-scale load transfer mechanism under different loading conditions is the mechanical response of the macro-scale properties of the asphalt mixtures. So, it is important to carry out further relevant studies to investigate load transfer mechanisms of asphalt mixtures and establish the corresponding evaluation system of the load transfer mechanism, which can provide a theoretical basis for explaining the macro-mechanical properties from the meso-scale mechanical mechanism.

(4) To date, systematic evaluation methods on the load transfer mechanism of asphalt mixtures are not well developed. These should be considered to efficiently obtain how the load transfer mechanism functions during the actual service of asphalt mixture through the analysis algorithm. Further, a reasonable evaluation system of the load transfer mechanism should be established in order to realize the effective evaluation of the actual road structure’s load-bearing capacity.

## 6. Recommendations

Extensive research has been carried out in the past to study the load transfer mechanisms of asphalt mixtures. The following points enlist the recommendations for future studies.

(1) The X-ray CT and DIP and numerical simulation is highly recommended to be used for investigating load transfer mechanism of asphalt mixtures.

(2) The contact structure is a complex topological structure where contact forces are transferred at various nodes to form force chains. The load transfer (contact, contact force, and force chain) mathematical model can be established according to the statistics method and graph method. Meanwhile, the optimal load-bearing structure of asphalt mixture can be quantified and analyzed by using the topology theory. It can provide the theoretical guidance for the mixture design.

(3) It is strongly recommended that several evaluation indicators (corresponding to contact, contact force, and force chain) are used in combination to characterize load transfer mechanisms.

(4) Based on the quantitative definition of the load transfer structure and characteristics, a series of studies should be conducted to explore the mechanism of the relationship between load transfer characteristics and performance. Numerical simulation experiments are carried out to explore the load transfer mechanism, and corresponding laboratory experiments are carried out to explore the macro-scale performance, so as to establish the mathematical model between the load transfer mechanism characterization indicator and macro-scale performance.

## Figures and Tables

**Table 1:** Test methods for characterizing load transfer mechanisms.

No.	Method	Main Features	Obtain	Studies
**Laboratory test method**				
1	Charge coupled device camera (CCD) or Computed tomography (CT) + DIP	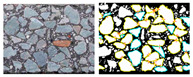 [8]	Contact number; contact chain.	[8,17,37]
2	Photoelastic experiment	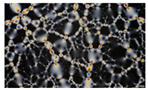	Contact number; contact chain; force chain.	[38,39,40,41]
3	Digital speckle correlation (DSC)	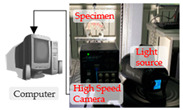	Strain state.	[42,43]
4	Intelligent aggregate (20 mm)	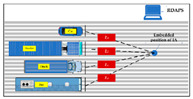 [44]	Stress at one point.	[44]
5	SmartRock	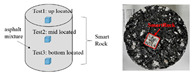 [45]	Change of contact structure; stress at one point.	[33,45]
6	Intelligent Attitude Aggregate (IAA).	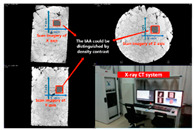 [46]	Change of contact structure.	[46]
7	Indentation test	--	Contact force.	[9,47]
8	Aggregate contact device (ACD)	--	Contact structure.	[48]
9	Magnetic Resonance Elastography (MRE)	--	Contact structure.	[49]
**Simulation test method**				
9	Discrete element method (DEM)	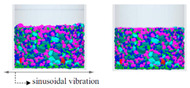 [5]	Contact number; contact chain; contact force; force chain.	[5,7,28]
10	Finite element method (FEM)	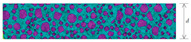 [50]	Contact chain; force chain.	[2,3,50,51]
11	DIP and numerical simulation	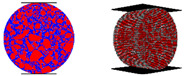 [52]	Contact number; contact chain; contact force; force chain.	[52,53]

**Table 2:** Typical indicators characterizing the load transfer mechanism.

Classification		Schematic Diagram	Indicators	References
Contact characteristics	Contact number	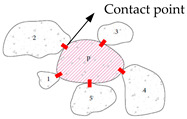 [37]	$n_{s}=\frac{\sum_{i=1}^{g}n_{s,i}}{g}$ ^1^	[21,37]
	Contact orientation		$\theta =\frac{\sum_{k=1}^{N}\left|\theta_{k}\right|}{N}$ ^2^ $\Delta =\frac{100}{N}\sqrt{{\left(\sum_{k=1}^{N}\sin2\theta_{k}\right)}^{2}+{\left(\sum_{k=1}^{N}\cos2\theta_{k}\right)}^{2}}$ ^3^	
	Contact angle	--	$\theta_{c}=\frac{180}{{\langle Z\rangle }^{\prime }}$ ^4^	[64]
	Contact chain	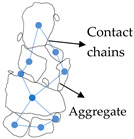	$E_{chain_{i}}=\sum_{path}\frac{h_{j}}{\sum_{path}h_{j}}E_{path_{j}}$ ^5^ $E_{skeleton}=\sum_{chain}\frac{\upsilon_{agg\_chain_{k}}}{c_{V}V_{specimen}}E_{chain_{k}}$ ^6^	[4]
	Contact distance	--	The minimum distance between one aggregate and its neighboring aggregates	[69]
	Contact length	--	$\bar{L_{c}}=\frac{1}{N_{c}}\sum_{i,j\neq i}^{N_{c}}l_{ij}$ ^7^	[17]
Contact force characteristics	Contact force	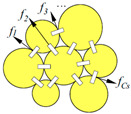 [5]	$\bar{F_{s}}=\frac{{\sum_{k=1}^{C_{s}}f_{k}}_{}}{C_{s}}$ ^8^ $P_{s}=\frac{\bar{F_{s_{}}}}{\sum_{i=1}^{l}\bar{F_{s_{i}}}}$ ^9^	[5,7]
	Unbalanced contact force	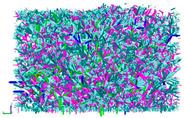 Color line: contact force	$\left\{ \begin{array}{l} F_{tavg}=\frac{\sum_{i=1}^{m}\left(\sum_{j=1}^{n_{j}}F_{ij}\right)}{\sum_{i=1}^{m}n_{i}}\\ f_{ti>tavg}=\frac{\sum_{j=1}^{n_{i}}\left(F_{ij}>F_{tavg}\right)}{\sum_{i=1}^{m}\left(\sum_{j=1}^{n_{i}}F_{ij}>F_{tavg}\right)}\\ f_{ti\leq tavg}=\frac{\sum_{j=1}^{n_{i}}\left(F_{ij}\leq F_{tavg}\right)}{\sum_{i=1}^{m}\left(\sum_{j=1}^{n_{i}}F_{ij}\leq F_{tavg}\right)} \end{array}\right.$ ^10^	[79]
	Contact force probability distribution	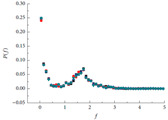 [23]	$P(f)=a(1-b\exp^{-cf^{2}})\exp^{-df^{}}$ ^11^	[22,23]
Force chain	Force chain number	--	$R_{MFC}=\frac{S_{MFC}}{S_{FC}}\times 100\%$ ^12^	[13,28]
	Force chain length		$\bar{l}=\frac{\sum_{i=1}^{N_{1}}l_{1i}+\sum_{i=1}^{N_{3}}l_{3i}}{N_{1}+N_{3}}$ ^13^	
	Force chain alignment coefficient		$\delta_{i}=1-\frac{\sum_{p=1}^{M}\alpha_{p}}{90^{{}^{\circ}}\times M}$ ^14^	
	Force chain direction angle	--	$\theta =\arccos\left(\frac{x_{B}-x_{A}}{\sqrt{{\left(x_{B}-x_{A}\right)}^{2}+{\left(y_{B}-y_{A}\right)}^{2}}}\right)$ ^15^	

^1^ Where *n_s_* is the contact number; *g* represents the total particle numbers of *A_s_*; *n_s_*, *I* is the contact number of the *i_th_* particle of *A_s_*. ^2,3^ Where *θ* is the average angle of inclination; Δ is the vector magnitude; $\theta_{k}$ is the angle between the horizontal axis and the major axis of an individual particle or the orientation of an individual contact line in one section; *N* is the total number of aggregates or contact lines in one section. ^4^ Where *θ_c_* is the contact angle; <*Z*> represents the properties of materials, such as elastic modulus, Poisson’s ratio, and gradation et al. ^5,6^ Where *E_chaini_* is the morphology of the contact chain; *h_j_* is the height of the bounding box of *path_j_*; *E_skeleton_* is the morphology of the contact skeleton; $\upsilon_{agg\_chain_{k}}$ is the volume sum of aggregates involved in *chain_k_*; *V_specimen_* is the volume of the specimen; *c_V_* is the coefficient. ^7^ Where $\bar{L_{c}}$ is the average contact length; *l*_ij_ is the length of the contact chain between coarse aggregates *i* and *j*; *Nc* is the quantity of coarse aggregates in the contact chain. ^8,9^ Where $\bar{F_{s}}$ is the average contact force of size s particles; *f_k_* is the contact force at contact point *k*; *C_s_* is the total contact number of size *s* particles; *l* is the size *l* particles; *P_s_* is the load-bearing contributions of size *s* particles. ^10^ Where *F_tavg_* is the total average force; *F_ij_* is the contact force; *f_ti_*>*t_avg_* is the proportion of contact force larger than the total average force in one sieve size to the contact force larger than the total average force in whole sieve sizes; *f_ti_*≤*t_avg_* is the proportion of contact force smaller than the total average force in one sieve size to the contact force smaller than the total average force in whole sieve sizes. ^11^ Where *P*(*f*) is the probability distribution of force chains; *f* is the ratio of the normal or shear contact force to the mean normal contact force; *a*, *b*, *c*, and *d* are the fitted parameters. ^12^ Where *S_MFC_* the asphalt mixture total number; *S_FC_* is the total number of force chains; *S_MFC_* is the total number of main force chains. ^13^ Where $\bar{l}$ is the average length of main force chain; *l_1i_* represents the length of *i*th I type force chains; *l_3i_* represents the length of *i*th III type force chains; *N_1_* and *N_3_* are the I and III type of force chain number, respectively. ^14^ Where $\delta_{i}$ is the main force chains alignment coefficient; *α_p_* represents the *p*th angle between adjacent normal directions of the *i*th *MFC*; *M* is the total number of adjacent contacts. ^15^ Where *θ* is the force chain direction angle; (*x_A_*, *y_A_*) and (*x_B_*, *y_B_*) are location coordinates of aggregates *A* and *B*, respectively.

## Data Availability

Not applicable.
